# Prospective Relationship of Low Cardiovascular Risk Factor Profile at Younger Ages to Ankle-Brachial Index: 39-Year Follow-Up—The Chicago Healthy Aging Study

**DOI:** 10.1161/JAHA.112.001545

**Published:** 2012-12-19

**Authors:** Thanh-Huyen T. Vu, Jeremiah Stamler, Kiang Liu, Mary M. McDermott, Donald M. Lloyd-Jones, Amber Pirzada, Daniel B. Garside, Martha L. Daviglus

**Affiliations:** Department of Preventive Medicine, Feinberg School of Medicine, Northwestern University, Chicago, IL (T.-H.T.V., J.S., K.L., M.M.M., D.M.L.-J., A.P., D.B.G., M.L.D.); Department of Medicine, Feinberg School of Medicine, Northwestern University, Chicago, IL (M.M.M., D.M.L.-J.)

**Keywords:** aging, atherosclerosis, cardiovascular disease, peripheral artery disease, risk factors

## Abstract

**Background:**

Data are sparse regarding the long-term association of favorable levels of all major cardiovascular disease risk factors (RFs) (ie, low risk [LR]) with ankle-brachial index (ABI).

**Methods and Results:**

In 2007–2010, the Chicago Healthy Aging Study reexamined a subset of participants aged 65 to 84 years from the Chicago Heart Association Detection Project in Industry cohort (baseline examination, 1967–1973). RF groups were defined as LR (untreated blood pressure ≤120/≤80 mm Hg, untreated serum cholesterol <200 mg/dL, body mass index <25 kg/m^2^, not smoking, no diabetes) or as 0 RFs, 1 RF, or 2+ RFs based on the presence of blood pressure ≥140/≥90 mm Hg or receiving treatment, serum cholesterol ≥240 mg/dL or receiving treatment, body mass index ≥30 kg/m^2^, smoking, or diabetes. ABI at follow-up was categorized as indicating PAD present (≤0.90), as borderline PAD (0.91 to 0.99), or as normal (1.00 to 1.40). We included 1346 participants with ABI ≤1.40. After multivariable adjustment, the presence of fewer baseline RFs was associated with a lower likelihood of PAD at 39-year follow-up (*P* for trend is <0.001). Odds ratios (95% CIs) for PAD in persons with LR, 0 RFs, or 1 RF compared with those with 2+ RFs were 0.14 (0.05 to 0.44), 0.28 (0.13 to 0.59), and 0.33 (0.16 to 0.65), respectively; findings were similar for borderline PAD (*P* for trend is 0.005). The association was mainly due to baseline smoking status, cholesterol, and diabetes. Remaining free of adverse RFs or improving RF status over time was also associated with PAD.

**Conclusions:**

LR profile in younger adulthood (ages 25 to 45) is associated with the lowest prevalence of PAD and borderline PAD 39 years later.

## Introduction

Peripheral arterial disease (PAD), a manifestation of systemic atherosclerosis, currently affects ≍12% to 20% of American men and women aged 65 years and older^1^ and costs the United States >$21 billion annually.^2^ Studies have shown that a low ankle-brachial index (ABI) is validated as a sensitive and specific marker for the presence of PAD^3,4^ and is associated with an increased risk of cardiovascular disease (CVD) morbidity and mortality and impaired quality of life.^3,5–13^

There is substantial evidence that having favorable levels of all major CVD risk factors (RFs) (ie, being at low risk [LR]) in younger adulthood is associated with reduced long-term CVD morbidity and mortality, lower healthcare costs, better quality of life, and less subclinical atherosclerosis.^14–25^ However, there has been little research linking CVD risk status in younger adulthood with ABI later in life. Some studies have documented the benefits of favorable levels of major CVD RFs (ie, smoking status, blood pressure [BP], serum cholesterol, and diabetes) when considered singly on favorable ABI.^26–31^ However, to our knowledge, whether a favorable CVD risk profile at younger adult ages is associated with a more favorable ABI later in life has not been previously examined. Establishing this association may encourage public health efforts to accomplish a progressive increase in the prevalence of LR, as recommended by the American Heart Association and Healthy People 2020,^32,33^ which aims to reduce the nation's burden of cardiovascular morbidity and mortality.

We addressed this question using data on 970 men and 376 women from the Chicago Healthy Aging Study (CHAS). Participants' risk profiles were ascertained in young adulthood/early middle age (1967–1973, at ages 25 to 45), and ABI was assessed 39 years later (2007–2010, at ages 65 to 84). We also assessed whether the association of baseline RF status with subsequent ABI levels (if any) persists regardless of current RF status. Moreover, we examined the predictive role of remaining free of all adverse cardiovascular RFs or of improved RF status over time in ABI levels at follow-up.

## Methods

### The Chicago Healthy Aging Study

CHAS is a study of a subset of participants from the Chicago Heart Association Detection Project in Industry (CHA), a public health program and prospective epidemiologic study conducted in 1967–1973 to identify high-risk adults in workplaces throughout the Chicago, Ill, area. Details of the CHA study have been published.^34^

There were 11 908 potential CHAS participants based on criteria as follows: CHA survivors, aged 65 to 84 years during 2007–2010, and free of major ECG abnormalities or myocardial infarction (MI) at the CHA examination (baseline). We used stratified sampling method to recruit CHAS participants based on their baseline RF profile (LR and not LR). Fifty-nine percent (n=7090) of names (988 LR and 6102 not LR) were randomly selected for contact by mail or telephone. We successfully contacted 2799 persons during 2007–2010, but 1404 persons refused to participate in CHAS, which provided a participation rate of 49.8%. The final CHAS sample included 1395 participants (28% women, 9.3% African American, 2.5% Hispanic or Asian, 19.5% baseline LR). LR participants were oversampled to obtain adequate numbers for between-group comparisons.

### Exclusions

Of 1395 CHAS participants examined at the clinic, we excluded 25 because of missing data on ABI (ankle or arm systolic BP [SBP] could not be measured). Individuals with ABI >1.40 in either leg were also excluded (n=24), because possible medial arterial calcification or partial or complete incompressibility of blood vessels made it difficult to accurately measure the lower-extremity pressure.^35^ Thus, the final sample for main analyses consisted of 1346 CHAS participants.

For analyses involving follow-up RF status, participants with missing follow-up RF values were also excluded (n=6), yielding 1340 participants.

### Measurement of Baseline RFs and Other Characteristics

Details of the measurements of RFs and other characteristics at baseline have been published.^36^ Briefly, trained staff measured participants' height, weight, supine BP, plasma glucose, and serum cholesterol using standardized collection methods. Body mass index (BMI) was calculated as weight in kilograms per height in meter squared. A single supine BP measurement was obtained using a standard mercury sphygmomanometer. Serum total cholesterol was measured by the Levine–Zak method.^37^ Resting ECGs were obtained and classified as showing minor (eg, nonspecific ST or T-wave abnormalities) or major (eg, major Q-wave or ventricular hypertrophy) abnormalities using criteria from the Pooling Project.^38^ Participants completed a questionnaire about demographic characteristics, smoking history, medical diagnoses, and treatments.^36,37^

### Measurement of Follow-up RFs

Standardized questionnaires were used to obtain information on smoking history, medical history of high BP or high serum cholesterol, and presence or absence of diabetes and MI. Height and weight were measured with participants wearing light clothing without shoes. Protocols and instruments were similar to those used in the Multi-Ethnic Study of Atherosclerosis (MESA) and the Coronary Artery Risk Development In young Adults (CARDIA) study.^39,40^ With participants seated, BP was measured 3 times using an automatic sphygmomanometer (Omron HEM-907 XL, Omron Healthcare, Inc., Bannockburn, IL).^40^ The average of the second and third measurements was used in the analyses. Total cholesterol and glucose levels were measured with 12-hour fasting blood samples. Diabetes was defined as a fasting glucose level >126 mg/dL or use of antihyperglycemic medication.^41^ A standard 12-lead ECG was obtained with the patient in a supine or semirecumbent position. ECGs were transmitted to the ECG Reading Center at Wake Forest University (Winston-Salem, NC), and criteria from the Minnesota Coding Center (Minneapolis, MN) were used for diagnostic classification of minor or major ECG abnormalities at the follow-up examination.^42^

### Definition of Risk Status

LR was defined as having all of the following: SBP/diastolic BP (DBP) ≤120/≤80 mm Hg and not taking antihypertensive medication, serum total cholesterol <200 mg/dL and not taking cholesterol-lowering medication, not being overweight or obese (BMI <25 kg/m^2^), no diabetes, and not smoking. Participants who were not LR were classified as having 0 RFs of a high level but ≥1 RF of an unfavorable level (0 RFs) (ie, SBP 121 to 139 mm Hg or DBP 81 to 89 mm Hg and not taking antihypertensive medication; serum cholesterol 200 to 239 mg/dL and not taking cholesterol-lowering medication; BMI 25.0 to 29.9 kg/m^2^) or as having only 1 RF of a high level (1 RF) or ≥2 RFs of a high level (2+ RFs) (SBP ≥140 or DBP ≥90 mm Hg or taking antihypertensive medication, serum cholesterol ≥240 mg/dL or taking cholesterol-lowering medication, BMI ≥30 kg/m^2^, current smoker, or having diabetes).

### ABI Measurement

ABI is the ratio of ankle SBP to arm SBP, computed separately for each leg. To compute ABI, a hand-held Doppler instrument with a 5-MHz probe (Nicolet Vascular, Golden, Colo) was used to obtain SBPs in the left and right brachial, dorsalis pedis, and posterior tibial arteries. Each ankle pressure was measured twice.^12^ Brachial artery pressures were averaged to obtain the ABI denominator. However, when the 2 brachial artery pressures differed by ≥10 mm Hg, the higher pressure was used as the denominator. For each lower extremity, the higher of the 2 pressures (dorsalis pedis or posterior tibial) was used as the ABI numerator. The lower of the 2 leg ABIs was used in the analyses. ABI was categorized as the presence of PAD (ABI <0.90), borderline PAD (0.91 to 0.99), or normal ABI (1.00 to 1.40), as defined in the 2011 ACCF/AHA focused update of the guideline for the management of patients with peripheral artery disease of the American College of Cardiology Foundation/American Heart Association Task Force on Practice Guidelines.^43^

All examination procedures were performed by trained and certified staff. The study was approved by the Northwestern University Institutional Review Board, and signed informed consent was obtained from all participants.

### Data Analyses

Descriptive characteristics were compared across the 4 baseline risk categories (ie, LR, 0 RFs, 1 RF, and 2+ RFs) using F tests for continuous variables or χ^2^ tests for binary variables. Age-, sex-, and race-adjusted prevalence rates of PAD and borderline PAD were computed for each risk category using general linear models, and the 4 RF categories were compared using logistic regression models, with the 2+ RF group designated as the reference group. Linear trend across the risk categories was also tested with logistic regression models.

Multinomial logistic regression models were used to examine the association of baseline LR status with follow-up prevalence of PAD/borderline PAD, using normal ABI (1.0≤ABI≤1.4) as the reference category. Baseline RF profile was coded as 1 representing LR; 2, 0 RFs; 3, 1 RF; and 4, 2+ RFs, and then was used as a continuous variable to test for trends across RF strata. Analyses were adjusted for baseline age, race, sex, and education attainment (model 1). Next, analyses were conducted with follow-up RF status included in the model to assess whether baseline RF status was associated with subsequent risk of PAD/borderline PAD independent of follow-up RF status. Because of the small proportion of LR persons at follow-up (n=28; 2% of the study cohort), the follow-up LR group was combined with the follow-up 0 RFs group in the analysis. In addition, because CHA participants with major ECG abnormalities or a history of MI at baseline were excluded from the study, these 2 factors were not included in the risk status definition. Therefore, the presence of follow-up MI or major ECG abnormalities was also adjusted in the model involving follow-up RF status (model 2). A model similar to model 2 was also used to assess the predictive role of remaining free of all adverse cardiovascular RFs or improved RF status over time in ABI levels at follow-up.

Models were repeated in sensitivity analyses using a low-normal ABI (1.00 to 1.09) as an additional ABI category and using 1.30 as the upper limit of normal for ABI.^3,12,13^

Models substituting individual RFs for the combined RF status groups were used to examine the ABI predictive value of each RF separately. Analyses stratified by sex were performed to assess possible effects of sex on the association. An interaction term for the LR profile and sex was also used.

All analyses were conducted using SAS statistical software version 9.2 (SAS Institute Inc, Cary, NC).

## Results

Of the 1346 CHAS participants with ABI <1.40 at follow-up (27.9% women; 88.3% non-Hispanic white, 9.3% African American, 2.4% Hispanic or Asian; baseline mean age 32.8 years [SD 4.6 years], current mean age 71.3 years [SD 4.6 years]), 19.4% [n=261] were LR and 15.1% [n=203] had ≥2 adverse RFs at baseline.

Characteristics of participants stratified by baseline RF category are shown in Table 1. LR participants tended to be women, white, and better educated than those in the other groups. Smoking and hypertension were more prevalent than other RFs at baseline. For example, among participants with 2+ RFs at baseline, 74.4% were current smokers and 84.2% had SBP/DBP ≥140/90 mm Hg or were receiving antihypertensive medication, whereas ≍25.1% were obese, 34.5% had a serum total cholesterol level ≥240 or were receiving cholesterol-lowering medication, and 3% had diabetes. Baseline LR participants were more likely to be RF free at follow-up (22.4%) than were those of the other baseline RF groups. In contrast, they are less likely to have follow-up MI or major ECG abnormalities (21.6%).

**Table 1.** Selected Baseline (1967–1973) and Follow-up (2007–2010) Characteristics, All Participants, and by Baseline RF Status

		Baseline RF Status				
			
Variable	All	LR*	0 RFs†	1 RF Only‡	2+ RFs‡	*P*‖
No. of people	1346	261	431	451	203	

Baseline characteristics						

Age, mean (SD), y	32.8 (4.6)	32.2 (5.0)	32.8 (4.5)	33.2 (4.5)	32.7 (4.4)	0.054

Female, n (%)	376 (27.9)	134 (51.3)	91 (21.1)	111 (24.6)	40 (19.7)	<0.001

Race, n (%)						

Black	125 (9.3)	22 (8.4)	30 (7.0)	45 (10.0)	28 (13.8)	0.177
	
Non-Hispanic white	1189 (88.3)	231 (88.5)	392 (91.0)	396 (87.8)	170 (83.7)	
	
Hispanic or Asian	32 (2.4)	8 (3.1)	9 (2.1)	10 (2.2)	5 (2.5)	

Education, mean (SD), y	14.8 (2.3)	14.8 (2.3)	15.2 (2.3)	14.6 (2.4)	14.4 (2.4)	<0.001

Smoking status, n (%)						

Never smoker	608 (45.2)	166 (63.6)	245 (56.8)	170 (37.7)	27 (13.3)	

Former smoker	413 (30.7)	95 (36.4)	186 (43.2)	107 (23.7)	25 (12.3)	

Current smoker	325 (24.2)	0 (0.0)	0 (0.0)	174 (38.6)	151 (74.4)	

BMI, mean (SD), kg/m^2^	24.9 (3.4)	22.1 (2.0)	25.3 (2.5)	25.1 (3.4)	27.2 (4.0)	

BMI ≥30.0 kg/m^2^, n (%)	84 (6.2)	0.0	0.0	33 (7.3)	51 (25.1)	

SBP, mean (SD), mm Hg	127.2 (15.0)	113.7 (6.2)	122.8 (8.6)	132.0 (15.4)	143.0 (13.3)	

DBP, mean (SD), mm Hg	75.4 (9.8)	68.9 (7.4)	73.4 (7.5)	77.7 (10.0)	83.2 (9.9)	

Hypertension,‡ n (%)	383 (28.5)	0.0	0.0	212 (47.0)	171 (84.2)	

Serum cholesterol, mean (SD), mg/dL	187.8 (35.5)	165.8 (21.3)	185.4 (27.8)	190.4 (35.4)	215.2 (44.2)	

Hypercholesterolemia,‡ n (%)	99 (7.4)	0.0	0.0	29 (6.4)	70 (34.5)	

Diabetes mellitus, n (%)	9 (0.7)	0.0	0.0	3 (0.7)	6 (3.0)	

Follow-up RF status,‡ n (%)						

0 RFs§	178 (13.3)	58 (22.4)	60 (14.0)	48 (10.7)	12 (6.0)	<0.001
	
1 RF only	344 (25.7)	85 (32.8)	114 (26.5)	115 (25.6)	30 (14.9)	
	
2+ RFs	818 (61.0)	116 (44.8)	256 (59.5)	287 (63.8)	159 (79.1)	

Follow-up MI/major ECG abnormalities, n (%)	409 (30.5)	56 (21.6)	128 (29.8)	150 (33.3)	75 (37.3)	0.001

RF indicates risk factor; LR, low risk; BMI, body mass index; SBP, systolic blood pressure; DBP, diastolic blood pressure.

* Favorable level of all major CVD RFs (BP ≤120/≤80 mm Hg and no antihypertensive medication, serum cholesterol <200 mg/dL and no lipid-lowering medication, not smoking, BMI <25 kg/m^2^, no diabetes).

† Unfavorable/borderline BP or serum total cholesterol, not smoking, BMI 25.0 to 29.9 kg/m^2^, no diabetes.

‡ High SBP/DBP (≥140/90) or using antihypertensive medication, serum total cholesterol ≥240 mg/dL or using lipid-lowering medication, smoking, BMI ≥30.0 kg/m^2^, diabetes.

§ Combined LR and 0 RFs groups at follow-up.

‖ *P* values for overall group comparisons based on χ^2^ or F test except for RF components.

**Figure 1.**
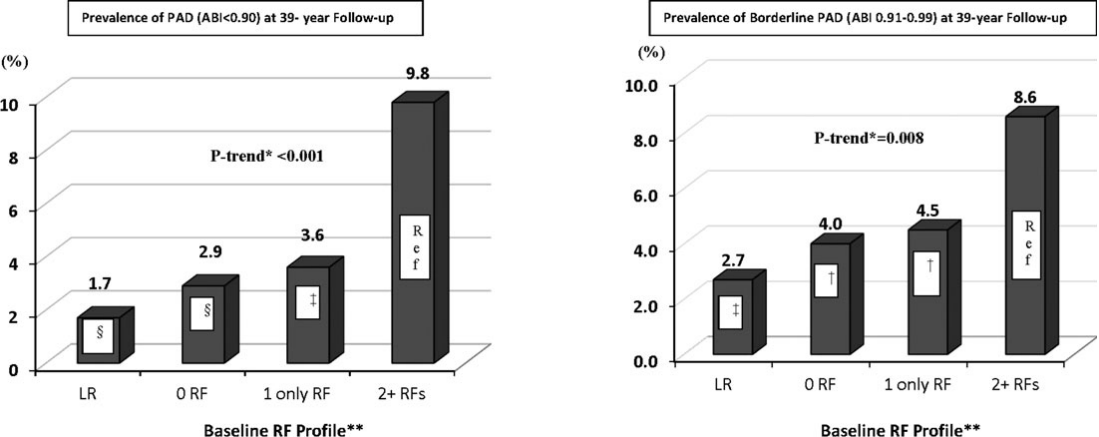
Age-, sex-, and race-adjusted prevalence of PAD or borderline PAD at follow-up examination (2007–2010) by baseline (1967–1973) RF status. *P value for trend across 4 RF groups. †*P*<0.05, ‡*P*<0.01, §*P*<0.001: *P* value for pairwise comparison with the 2+ RFs as reference group. **Baseline RF profile: LR, favorable level of all major CVD RFs (SBP/DBP ≤120/≤80 mm Hg and no antihypertensive medication, serum total cholesterol <200 mg/dL and no lipid-lowering medication, not smoking, BMI <25 kg/m^2^, and no diabetes); 0 RFs, borderline level of SBP/DBP or serum total cholesterol, not smoking, BMI 25.0 to 29.9 kg/m^2^, no diabetes; RF, high SBP/DBP (≥140/90) or using antihypertensive medication, serum total cholesterol ≥240 mg/dL or using lipid-lowering medication, smoking, BMI ≥30.0 kg/m^2^, or diabetes. PAD indicates peripheral arterial disease; RF, risk factor; LR, low risk; CVD, cardiovascular disease; SBP, systolic blood pressure; DBP, diastolic blood pressure; BMI, body mass index.

At follow-up, 3.9% (n=53) of participants had PAD and 4.6% (n=62) had borderline PAD. The age-, sex-, and race-adjusted prevalence of PAD at follow-up was lowest among the LR group and increased with the number of RFs, with the *P* value for trend across 4 RF groups <0.001 (Figure 1). Similarly, age-, sex-, and race-adjusted prevalence of borderline PAD was lowest for the LR group and increased with the number of RFs (*P* value for trend across 4 RF groups 0.008).

With adjustment for baseline age, sex, race, and education attainment, a more favorable baseline risk profile was associated with a substantially lower likelihood of PAD or borderline PAD at follow-up (β coefficient=0.63; *P* value for trend <0.001 for PAD versus normal ABI level, and β coefficient=0.40; *P* value for trend 0.005 for borderline versus normal ABI level). The odds ratio (OR) (95% CI) of having PAD and borderline PAD at follow-up in participants with baseline LR compared with those with baseline 2+ RFs were 0.14 (0.05 to 0.44) and 0.28 (0.12 to 0.66), respectively. The association of baseline RF profile with ABI level at follow-up remained significant with adjustment for follow-up RF profile and the presence of MI or major ECG abnormalities at follow-up in the model (β coefficient=0.59; *P* value for trend <0.001 for PAD versus normal ABI level, and β coefficient=0.34; *P* value for trend 0.021 for borderline versus normal ABI level) (Table 2). Furthermore, the OR (95% CI) of having PAD at follow-up in persons who remained free of all adverse cardiovascular RFs or improved their RF status over time compared with those whose risk status worsened or did not improve over time was 0.12 (0.02 to 0.90) (results not tabulated).

**Table 2.** Adjusted* ORs (95% CI) for PAD/Borderline PAD at Follow-up (2007–2010) by Baseline (1967–1973) RF Status

		ABI Level at Follow-up	
		
Baseline RF Group	N	PAD (≤0.90) vs Normal ABI (1.00 to 1.40)	Borderline PAD (0.91 to 0.99) vs Normal ABI (1.00 to 1.40)
Model 1*			

LR†	261	0.14 (0.05 to 0.44)	0.28 (0.12 to 0.66)

0 RFs‡	431	0.28 (0.13 to 0.59)	0.39 (0.19 to 0.79)

1 RF only§	451	0.33 (0.16 to 0.65)	0.43 (0.22 to 0.86)

≥2 RFs§	203	1.00	1.00

β Coefficient/*P* for trend¶		0.63/<0.001	0.40/0.005

Model 2*			

LR†	259	0.16 (0.04 to 0.57)	0.32 (0.13 to 0.81)

0 RFs‡	430	0.31 (0.14 to 0.69)	0.43 (0.21 to 0.90)

1 RF only§	450	0.38 (0.19 to 0.77)	0.46 (0.23 to 0.94)

≥2 RFs§	201	1.00	1.00

β Coefficient/*P* for trend¶		0.59/<0.001	0.34/0.021

OR indicates odds ratio; PAD, peripheral arterial disease; RF, risk factor; ABI, ankle-brachial index; LR, low risk; CVD, cardiovascular disease; BMI, body mass index; SBP, systolic blood pressure; DBP, diastolic blood pressure.

* Model 1 adjusted for baseline age, sex, race, and education attainment (n=1346). Model 2 adjusted for all variables from model 1 plus current RF status and presence of follow-up MI/major ECG abnormalities (n=1340).

† Favorable level of all major CVD RFs (SBP/DBP ≤120/≤80 mm Hg and no antihypertensive medication, serum total cholesterol <200 mg/dL and no lipid-lowering medication, not smoking, BMI <25 kg/m^2^, no diabetes).

‡ Borderline SBP/DBP or serum total cholesterol, not smoking, BMI 25.0 to 29.9 kg/m^2^, no diabetes.

§ High SBP/DBP (≥140/90) or using antihypertensive medication, serum total cholesterol ≥240 mg/dL or using lipid-lowering medication, smoking, BMI ≥30.0 kg/m^2^, or diabetes.

¶ *P* value for trend across 4 baseline RF groups based on Wald Chi-Square tests.

In sensitivity analyses using low-normal ABI (1.00 to 1.09) as an additional ABI category, no association of baseline risk profile with low-normal ABI was found, although the trend is still consistent (β coefficient=0.09; *P* value for trend = 0.233 for low-normal ABI [1.00 to 1.09] versus normal ABI level [1.10 to 1.40]). Furthermore, the use of 1.30 as the upper limit of normal for ABI yielded similar results compared with the upper limit of 1.40.

In analyses of the predictive value of each baseline RF considered separately for low ABI values, cigarette smoking, higher cholesterol levels, and diabetes were associated with lower subsequent ABI levels (Table 3). For example, ORs (95% CI) for PAD were 0.28 (0.14 to 0.58) for never smoking versus current smokers and 0.42 (0.17 to 0.99) for favorable cholesterol level versus cholesterol level ≥240 mg/dL or receiving cholesterol-lowering medication. Participants with diabetes at baseline were 7 times as likely (95% CI, 1.64 to 33.26) to have developed borderline PAD at follow-up as were those who did not have no diabetes at baseline; the predictive role of baseline diabetes for PAD versus normal ABI level could not be evaluated because no participants with PAD at follow-up had diabetes at baseline.

**Table 3.** Adjusted* ORs (95% CI) for PAD/Borderline PAD at Follow-up (2007–2010) by Individual Baseline RFs (1967–1973)

	ABI Level at Follow-up	
	
Baseline RF	PAD (≤0.90) vs Normal ABI (1.00 to 1.40)	Borderline PAD (0.91 to 0.99) vs Normal ABI (1.00 to 1.40)
Smoking status		

Never smoker	**0.28 (0.14 to 0.58)**‖	**0.35 (0.18 to 0.66)**§

Former smoker	**0.41 (0.20 to 0.83)**‡	0.53 (0.27 to 1.03)

Current smoker (reference)	1.00	1.00

BP level/medication use (medications)		

≤120/80 mm Hg, no medications	0.91 (0.44 to 1.90)	1.10 (0.56 to 2.18)

>120/80 and ≤140/90, no medications	1.09 (0.54 to 2.18)	1.19 (0.58 to 2.48)

≥140/90 or receiving medications (reference)	1.00	1.00

Cholesterol level/medication use		

<200 mg/dL, no medications	**0.42 (0.17 to 0.99**)†	**0.37 (0.17 to 0.83)**†

≥200 and <240, no medications	0.76 (0.31 to 1.86)	0.49 (0.21 to 1.17)

≥240 or receiving medications (reference)	1.00	1.00

BMI, kg/m^2^		

<25	0.36 (0.13 to 1.03)	2.02 (0.54 to 7.52)

≥25 and <30	0.82 (0.32 to 2.11)	1.33 (0.36 to 4.94)

≥30 (Reference)	1.00	1.00

Diabetes	…†	**7.39 (1.64 to 33.26)**§

OR indicates odds ratio; PAD, peripheral arterial disease; RF, risk factor; ABI, ankle-brachial index; BP, blood pressure; BMI, body mass index.

* Adjusted for baseline age, sex, race, and education attainment.

† The predictive role of baseline diabetes for PAD vs normal ABI level cannot be evaluated because no participants with PAD at follow-up had diabetes at baseline.

‡ *P*<0.05,

§ *P*<0.01,

‖ *P*<0.001: *P*-value for pair-wise comparison with the reference group.

In sex-specific analyses, results were similar for men and women. ORs (95% CI) for the presence of PAD in persons with baseline LR were 0.17 (0.04 to 0.78) for men and 0.11 (0.02 to 0.57) for women, compared with the sex-specific estimates in the baseline 2+ RFs stratum (Table 4). There were no significant interactions between sex and baseline RF profile for associations with PAD or borderline PAD at follow-up.

**Table 4.** Sex-Specific Adjusted* ORs (95% CI) for PAD/Borderline PAD at Follow-up (2007–2010) by Baseline (1967–1973) RF Status

	Men			Women		
		
		ABI Level at Follow-up			ABI Level at Follow-up	
				
Baseline RF Group	N	PAD (≤0.90) vs Normal ABI (1.00 to 1.40)	Borderline PAD (0.91 to 0.99) vs Normal ABI (1.00 to 1.40)	N	PAD (≤0.90) vs Normal ABI (1.00 to 1.40)	Borderline PAD (0.91 to 0.99) vs Normal ABI (1.00 to 1.40)
LR†	127	**0.17 (0.04** to **0.78)**#	0.13 (0.02 to 1.00)	134	**0.11 (0.02** to 0.57)**	**0.29 (0.09** to **0.93)**#

0 RFs§	340	**0.34 (0.14** to **0.81)**#	0.44 (0.17 to 1.14)	91	**0.13 (0.02** to **0.68)**#	0.39 (0.12 to 1.27)

Only 1 RF§	340	**0.28 (0.12** to 0.67)**	0.54 (0.23 to 1.27)	111	0.37 (0.11 to 1.24)	0.33 (0.10 to 1.06)

≥2 RFs§	163	1.00	1.00	40	1.00	1.00

β Coefficient/*P* trend‖		**0.55/0.008**	**0.50/0.019**		**0.81/0.004**	0.29/0.130

OR indicates odds ratio; PAD, peripheral arterial disease; RF, risk factor; ABI, ankle-brachial index; LR, low risk; CVD, cardiovascular disease; BMI, body mass index; SBP, systolic blood pressure; DBP, diastolic blood pressure.

* Adjusted for baseline age, race, and education attainment.

† Favorable level of all major CVD RFs (SBP/DBP ≤120/≤80 mm Hg and no antihypertensive medication, serum total cholesterol <200 mg/dL and no lipid-lowering medication, not smoking, BMI <25 kg/m^2^, and no diabetes).

‡ Borderline of SBP/DBP or serum total cholesterol, not smoking, BMI 25.0 to 29.9 kg/m^2^, no diabetes.

§ High SBP/DBP (≥140/90) or using antihypertensive medication, serum total cholesterol ≥240 mg/dL or using lipid-lowering medication, smoking, BMI ≥30.0 kg/m^2^, or diabetes.

‖ *P* values for trend across 4 baseline RF groups.

# *P*<0.05,

** *P*<0.01: *P*-value for pair-wise comparison with the reference group.

## Discussion

We found that persons in young adulthood/early middle age with a low coronary heart disease/CVD risk profile (favorable levels of all major RFs) had significantly lower odds of having PAD or borderline PAD 39 years later, compared with those with unfavorable or high RF levels in young adulthood/early middle age. This association was independent of baseline age, sex, race, education, and current RF status. Remaining free of all adverse RFs or improving RF status over time was also associated with lower odds of PAD.

To our knowledge, this study is the first to examine the long-term association of LR profile at younger age with PAD at older age in a general population of both men and women. Prior studies have documented substantial benefits of the LR profile in young adulthood and middle age for other health outcomes at older age, including increased longevity, lower CVD and total mortality, markedly lower long-term and lifetime risks of CVD and other chronic diseases, better health-related quality of life, and lower heathcare costs.^14–25^ There has also been substantial research on the association of PAD as measured by ABI and health outcomes. PAD is associated with increased risk of CVD morbidity and mortality, greater loss of mobility, and poorer quality of life.^3,5–12^ For example, in a meta-analysis, 10-year adjusted hazard ratios of CVD mortality in persons with PAD were 2.9 (95% CI, 2.3 to 3.7) for men and 3.0 (95% CI, 2.0 to 4.4) for women compared with those with a normal ABI level.^3^ In the MESA, the risk of CVD events with low ABI (ABI <1.0) was 1.8 times (*P*<0.001) higher than that with a normal ABI level (1.0≤ABI<1.4).^5^ Low ABI level was associated with higher incidence of mobility loss and impaired health-related quality of life.^12^ ABI therefore is widely considered to be not only a marker of PAD but also an important biomarker of global cardiovascular risk.^44^

To date, only a few population-based studies have focused on associations of CVD RFs and ABI. Findings from these studies indicate consistent and significant influences of several RFs on ABI, including smoking status, BP, cholesterol, and diabetes.^26–31^ These studies variously focused on the role of CVD RFs considered singly,^26–31^ examined associations only in very old population strata and only among minority men,^26^ had short-term follow-up,^27–29^ or examined only cross-sectional associations.^30,31^ For example, among 3450 ambulatory Japanese American men aged 71 to 93 years in the Honolulu Heart Program, RFs measured at baseline were predictive of low ABI level with 25 years of follow-up: OR of ABI <0.9 for those with cholesterol at the 80th percentile (5.59 mmol/L) compared with those at the 20th percentile (4.19 mmol/L) was 1.4; for hypertension versus normal BP, 1.8; and for current smokers versus nonsmokers, 2.9 (*P*<0.05 for all ORs).^26^ Among 50-year-old Danish men and women (N=666), male sex, BP, serum cholesterol, serum triglycerides, smoking, and heart rate measured at age 50 were significantly associated with signs of peripheral arteriostenosis and with ABI levels after 10-year follow-up.^27^

In the Cardiovascular Health Study with 2289 men and women aged 65 years or older having ABI measured in the same leg in 1992–1993 and 1998–1999, the ORs (95% CI) of incident lower-extremity arterial disease were 1.74 (1.02 to 2.96) for current smoking, 1.64 (1.18 to 2.28) for hypertension, 1.77 (1.14 to 2.76) for diabetes, 1.60 (1.03 to 2.51) for higher low-density lipoprotein cholesterol level, and 1.74 (1.05 to 2.89) for lipid-lowering medication use.^29^ These reports all lack information on possible long-term beneficial influence on ABI of having favorable levels of all major CVD RFs simultaneously in early adulthood. With a general population including both men and women, our current study benefited from the ability to examine long-term associations for combined and individual major CVD RFs from younger age (mean age 32 years) with ABI levels at older age (mean age 71 years).

Our current study yielded results consistent with findings reported by previous studies on the associations of individual major CVD RFs and ABI levels; baseline smoking status, serum cholesterol, and diabetes were powerful predictors of ABI level at 39 year follow-up.^26,27,29^ However, baseline BP did not play an important role. Although the PAD prevalence was lower in our study participants than in other older cohorts,^45,46^ this observation may be explained in that CHAS participants are healthier than the general population for several reasons: the original CHA participants were employees, which may make the CHA cohort healthier than the general population; those who participated in CHAS are more likely to be healthier than those who did not participate; and LR individuals in CHAS were oversampled. Furthermore, previous studies have reported inconsistent findings on the effects of sex on the association of LR status and ABI^27,30,31^; we did not find any significant sex differences in these associations.

This study has several strengths, such as its 39-year follow-up, with the ability to study low risk of coronary heart disease/CVD in young adulthood/early middle age and its effect on health in older age, in men and women from wide socioeconomic and ethnic spectra.^37^

Limitations include a lack of data on baseline or interim data on ABI (ie, only a single measurement of ABI at 39-year follow-up). As a result, we are unable to assess the progression of ABI over time. Because of the long interval between examinations, the matter of survival bias may be deemed relevant. However, individuals with higher RF burden at a younger age would have been more likely to die in the interim; these individuals would also have been more likely to develop PAD. Hence, if anything, we may be underestimating the strength of the LR profile association with ABI over the long term. In addition, early and sustained therapy such as lifestyle changes and pharmacological therapy may modify decades-long ABI risk, and we are also unable to address this issue here. Nevertheless, the favorable association of LR status at a younger age (mainly related to baseline smoking status, cholesterol, and diabetes) is still useful in the 21st century and may help inform therapy decisions for midlife patients.

In conclusion, in addition to substantial benefits of a favorable CVD risk profile as shown previously, the additional evidence here of the benefits of LR status further underscores the relevance of LR status at a younger age for long-term cardiovascular health. LR profile early in life is associated with lower risk of atherosclerosis in the extremities, as well as overall lower morbidity, mortality, and healthcare costs later in life. Preventive assessment of RFs early in life and public health initiatives to increase the population-wide proportion of LR persons are needed to prevent and delay the onset of CVD later in life. Recently, the American Heart Association developed its Strategic Impact Goal Through 2020,^32^ which aims to improve the cardiovascular health of all Americans by 20%. This health initiative helps achieve the goals of Healthy People 2020,^33^ the official national effort to attain high-quality, long lives free of preventable disease. Such efforts are highly relevant for the US society, especially because the prevalence of low risk is inordinately low.^47^
